# A combined radiomics and clinical model for preoperative differentiation of intrahepatic cholangiocarcinoma and intrahepatic bile duct stones with cholangitis: a machine learning approach

**DOI:** 10.3389/fonc.2025.1546940

**Published:** 2025-03-17

**Authors:** Hongwei Qian, Yanhua Huang, Yuxing Dong, Luohang Xu, Ruanchang Chen, Fangzheng Zhou, Difan Zhou, Jianhua Yu, Baochun Lu

**Affiliations:** ^1^ Department of Hepatobiliary and Pancreatic Surgery, Shaoxing People’s Hospital, Shaoxing, China; ^2^ Shaoxing Key Laboratory of Minimally Invasive Abdominal Surgery and Precise Treatment of Tumor, Shaoxing, China; ^3^ Department of Ultrasound, Shaoxing People’s Hospital, Shaoxing, China; ^4^ School of Medicine, Zhejiang University, Hangzhou, Zhejiang, China; ^5^ School of Medicine, Shaoxing University, Shaoxing, Zhejiang, China

**Keywords:** intrahepatic cholangiocarcinoma, intrahepatic bile duct stones, intrahepatic lithiasis, radiomics, nomogram

## Abstract

**Background:**

This study aimed to develop and validate a predictive model integrating radiomics features and clinical variables to differentiate intrahepatic bile duct stones with cholangitis (IBDS-IL) from intrahepatic cholangiocarcinoma (ICC) preoperatively, as accurate distinction is crucial for determining appropriate treatment strategies.

**Methods:**

A total of 169 patients (97 IBDS-IL and 72 ICC) who underwent surgical resection were retrospectively analyzed. Radiomics features were extracted from ultrasound images, and clinical variables with significant differences between groups were identified. Feature selection was performed using LASSO regression and recursive feature elimination (RFE). The radiomics model, clinical model, and combined model were constructed and evaluated using the area under the curve (AUC), calibration curves, decision curve analysis (DCA), and SHAP analysis.

**Results:**

The radiomics model achieved an AUC of 0.962, and the clinical model achieved an AUC of 0.861. The combined model, integrating the Radiomics Score with clinical variables, demonstrated the highest predictive performance with an AUC of 0.988, significantly outperforming the clinical model (*p* < 0.05). Calibration curves showed excellent agreement between predicted and observed outcomes, and the Hosmer-Lemeshow test confirmed a good model fit (*p* = 0.998). DCA revealed that the combined model provided the greatest clinical benefit across a wide range of threshold probabilities. SHAP analysis identified the Radiomics Score as the most significant contributor, complemented by abdominal pain and liver atrophy.

**Conclusion:**

The combined model integrating radiomics features and clinical data offers a powerful and reliable tool for preoperative differentiation of IBDS-IL and ICC. Its superior performance and clinical interpretability highlight its potential for improving diagnostic accuracy and guiding clinical decision-making. Further validation in larger, multicenter datasets is warranted to confirm its generalizability.

## 1 Introduction

Intrahepatic bile duct stones combined with intrahepatic lithiasis (IBDS-IL), and intrahepatic cholangiocarcinoma (ICC) are two significant conditions that pose diagnostic challenges in clinical practice (1–3). ICC is the second most common primary liver malignancy after hepatocellular carcinoma, and its global incidence is steadily increasing, with notable geographic variations. In particular, regions such as Thailand exhibit a higher incidence due to factors like parasitic infections and the high prevalence of IBDS-IL (4).

Patients with intrahepatic bile duct stones often develop cholangitis, a chronic inflammatory condition that can lead to localized liver atrophy and increase the risk of carcinogenesis (5). The risk factors for ICC are complex, but IBDS-IL has recently been identified as a strong risk factor (6). Several studies have shown that a considerable proportion of patients with IBDS-IL eventually develop cholangiocarcinoma, complicating the diagnostic process for clinicians (7). Accurate differentiation between cholangitis and ICC is essential for effective clinical management. Misdiagnosing ICC as benign cholangitis can delay treatment and lead to disease progression, while mistaking cholangitis for malignancy may result in unnecessary surgical interventions, such as performing lymphadenectomy inappropriately (8). This distinction plays a critical role in guiding appropriate treatment strategies and optimizing patient outcomes.

Current imaging modalities, particularly ultrasound, play a critical role in the initial assessment of patients with biliary symptoms. However, conventional ultrasound techniques often struggle to differentiate between cholangitis and ICC effectively, resulting in diagnostic uncertainty and potential treatment errors (9). Although computed tomography (CT) can provide useful imaging findings for ICC, classic CT features are observed in only a portion of cases, and the diagnostic accuracy for distinguishing IBDS-IL complicated by ICC remains low, typically ranging from 30% to 65% (10, 11).

In recent years, the emerging field of radiomics, which involves the quantitative extraction of high-throughput imaging features, has shown great potential (12, 13). By analyzing subtle imaging patterns that are difficult to detect with the naked eye, radiomics has demonstrated the ability to enhance the accuracy of disease diagnosis, pathological grading, prognosis evaluation, and treatment response prediction (14, 15). Although radiomics has achieved favorable outcomes in the clinical management of various cancers, there remains a lack of specific tools to distinguish IBDS-IL from ICC.

This study aims to develop and validate a radiomics-based model using ultrasound images for the preoperative identification of ICC among patients with IBDS-IL. By identifying key imaging features that differentiate cholangitis from ICC, we hope to advance non-invasive diagnostic approaches for hepatobiliary diseases and ultimately improve patient outcomes.

## 2 Materials and methods

### 2.1 Study population

Our study retrospectively analyzed patients who underwent liver resection and were pathologically diagnosed with IBDS-IL or ICC at our institution between September 2015 and September 2024. The inclusion criteria were: (1) Age ≥ 18 years; (2) abdominal ultrasound performed within two weeks before surgery; (3) postoperative pathological confirmation of IBDS-IL or ICC; (4) Patients and family consent to participate in the study. The exclusion criteria were: (1) incomplete pathological data; (2) incomplete clinical data; (3) missing or suboptimal quality ultrasound images. Ultimately, 169 patients were included in the study, consisting of 97 with IBDS-IL and 72 with ICC. The patients were randomly divided into a training group (118 individuals) and a validation group (51 individuals), ensuring both groups were representative and suitable for further investigations and analyses.

**Table 1** compares the overall clinical characteristics of IBDS-IL and ICC, as well as the clinical data of IBDS-IL and ICC within both the training and validation groups. Abdominal pain was defined as upper abdominal discomfort or pain reported by the patient during hospital visits, as documented in clinical records. Liver atrophy was assessed by experienced radiologists based on available imaging data and diagnosed according to morphological features such as reduced liver volume, irregular contour, and segmental atrophy.

**Table 1:** Demographic and clinical characteristics of patients.

Variables	IBDS-IL(n=97)	ICC(n=72)	*p*	Training Group(n=118)		*p*	Testing Group(n=51)		*p*
				IBDS-IL(n=67)	ICC(n=51)		IBDS-IL(n=30)	ICC(n=21)	
Age	62.79 ± 9.26	67.07 ± 9.3	0.004^*^	61.97 ± 9.67	67.62 ± 9.32	0.002^*^	64.72 ± 7.91	65.82 ± 9.15	0.656
BMI	22.32 ± 2.81	22.04 ± 3.09	0.546	22.26 ± 2.9	22.11 ± 3.1	0.792	22.45 ± 2.58	21.86 ± 3.05	0.47
AFP	2.23 ± 1.17	18.84 ± 106.73	0.13	2.26 ± 1.2	8.12 ± 29.43	0.107	2.16 ± 1.12	43.21 ± 185.62	0.249
CA199	13.82(6.32-31.74)	62.14(12.61-2761.52)	0.034^*^	16.64(7.9-33.74)	85.4(14.17-2761.52)	0.075	10.82(5.26-23.07)	49.56(14.36-306.91)	0.036^*^
CEA	2.15(1.62-3.45)	4.76(2.58-10.96)	0.004^*^	2.1(1.56-3.24)	4.84(2.89-9.36)	0.01^*^	2.78 ± 1.28	37.83 ± 98.6	0.067
CA125	12.2(9.15-16.54)	21.98(12.44-100.6)	0.009^*^	12.2(9.02-16.76)	24.21(13.88-105.62)	0.046^*^	12.17(10.0-16.02)	17.61(10.44-33.96)	0.073
ALT	38.0(20.1-125.7)	20.45(15.55-40.95)	0.008^*^	45.65(18.55-172.55)	19.75(15.55-36.55)	0.02^*^	82.17 ± 151.91	43.52 ± 54.73	0.27
AST	133.83 ± 367.1	52.22 ± 68.07	0.066	155.68 ± 419.41	54.93 ± 76.02	0.099	82.61 ± 185.87	46.05 ± 44.43	0.38
ALP	102.8(73.6-158.1)	119.3(86.0-217.5)	0.013^*^	139.31 ± 96.67	176.68 ± 168.21	0.134	109.9(88.4-153.4)	166.0(86.35-220.85)	0.031^*^
GGT	181.2 ± 253.08	214.98 ± 335.22	0.459	179.21 ± 268.33	159.06 ± 207.29	0.662	185.87 ± 213.04	342.07 ± 496.85	0.143
TBIL	25.01 ± 25.84	34.04 ± 92.37	0.364	26.24 ± 28.61	37.72 ± 106.46	0.402	22.14 ± 17.37	25.67 ± 45.43	0.709
DBIL	11.69 ± 18.64	19.61 ± 70.54	0.295	12.52 ± 20.93	22.96 ± 82.06	0.321	9.75 ± 11.37	11.99 ± 29.96	0.719
ALB	37.5 ± 5.78	37.78 ± 4.21	0.731	37.5 ± 5.91	38.27 ± 4.15	0.436	37.51 ± 5.46	36.67 ± 4.15	0.561
PT	12.48 ± 1.17	12.83 ± 1.4	0.079	12.36 ± 1.06	12.82 ± 1.51	0.057	12.77 ± 1.35	12.86 ± 1.08	0.792
INR	1.03 ± 0.1	1.04 ± 0.12	0.735	1.02 ± 0.08	1.04 ± 0.13	0.379	1.05 ± 0.12	1.03 ± 0.08	0.487
Gender			<0.05^*^			0.002^*^			0.097
Female	64	28		48	21		16	7	
Male	33	44		20	29		13	15	
Abdominal Pain			<0.05^*^			<0.05^*^			0.001^*^
No	18	52		11	36		7	16	
Yes	79	20		57	14		22	6	
Combined with Common Bile Duct Stones			<0.05^*^			<0.05^*^			0.246
No	48	58		30	41		18	17	
Yes	49	14		38	9		11	5	
Weight Loss in the Past 3 Months			0.831			0.826			1
No	96	71		67	49		29	22	
Yes	1	1		1	1		0	0	
Smoking			0.011^*^			0.045^*^			0.121
No	83	50		59	36		24	14	
Yes	14	22		9	14		5	8	
Drinking Alcohol			0.033^*^			0.006^*^			0.906
No	84	53		62	36		22	17	
Yes	13	19		6	14		7	5	
Diabetes			0.802			0.671			0.773
No	85	64		58	44		27	20	
Yes	12	8		10	6		2	2	
Hypertension			0.737			0.835			0.77
No	65	50		45	34		20	16	
Yes	32	22		23	16		9	6	
Hepatitis B			<0.05^*^			0.016^*^			0.007^*^
No	94	58		65	41		29	17	
Yes	3	14		3	9		0	5	
Combined with Other Tumors			0.001^*^			0.007^*^			0.017^*^
No	90	53		61	35		29	18	
Yes	7	19		7	15		0	4	
Family History of Tumors			0.831			0.826			1
No	96	71		67	49		29	22	
Yes	1	1		1	1		0	0	
Liver Atrophy			<0.05^*^			<0.05^*^			<0.05^*^
No	21	59		15	41		6	18	
Yes	76	13		53	9		23	4	
Liver Cirrhosis			0.014^*^			0.072			0.08
No	92	60		64	42		28	18	
Yes	5	12		4	8		1	4	
Fatty Liver			0.064			0.057			0.606
No	83	68		58	48		25	20	
Yes	14	4		10	2		4	2	

BMI, body mass index; AFP, alpha fetoprotein; CA199, cancer antigen 199; CEA, carcinoembryonic antigen; CA125, cancer antigen 125; ALT, alanine transaminase; AST, aspartate transaminase; ALP, alkaline phosphatase; GGT, Gamma-Glutamyl transferase; TBIL, total bilirubin; DBIL, directed bilirubin; ALB, albumin level; PT, prothrombin time; INR, international normalized ratio; *p<0.05.

**Figure 1:**
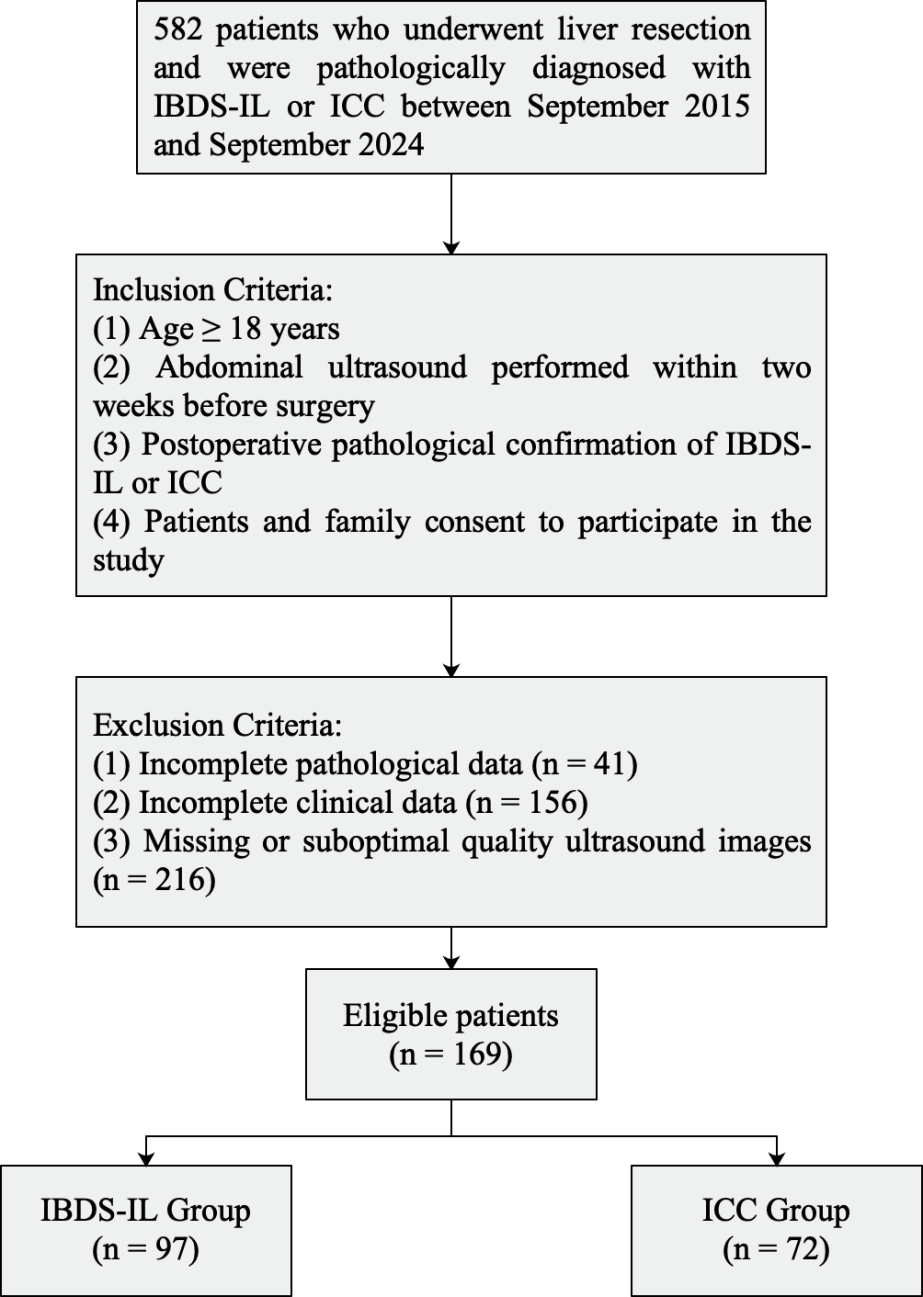
Flowchart of included and excluded patients.

A flowchart of the included and excluded patients is shown in **Figure 1**.

### 2.2 Image acquisition

All ultrasound examinations were performed by experienced radiologists following a standardized protocol to ensure consistency and reliability of the imaging data. Patients were positioned in either the supine or lateral decubitus position with their arms raised to fully expose the liver area for optimal imaging. A coupling gel was applied between the ultrasound probe and the skin to enhance sound wave transmission and minimize interference. After identifying the lesion with conventional 2D ultrasound, the images were adjusted to obtain the best view of the lesion. Multiple images were captured from different angles of the lesion for each patient, and all images were stored in digital imaging and communications in medicine (DICOM) format for subsequent analysis. Details of the ultrasound equipment used are provided in the **Supplementary Material**.

### 2.3 Image segmentation

**Figure 2:**
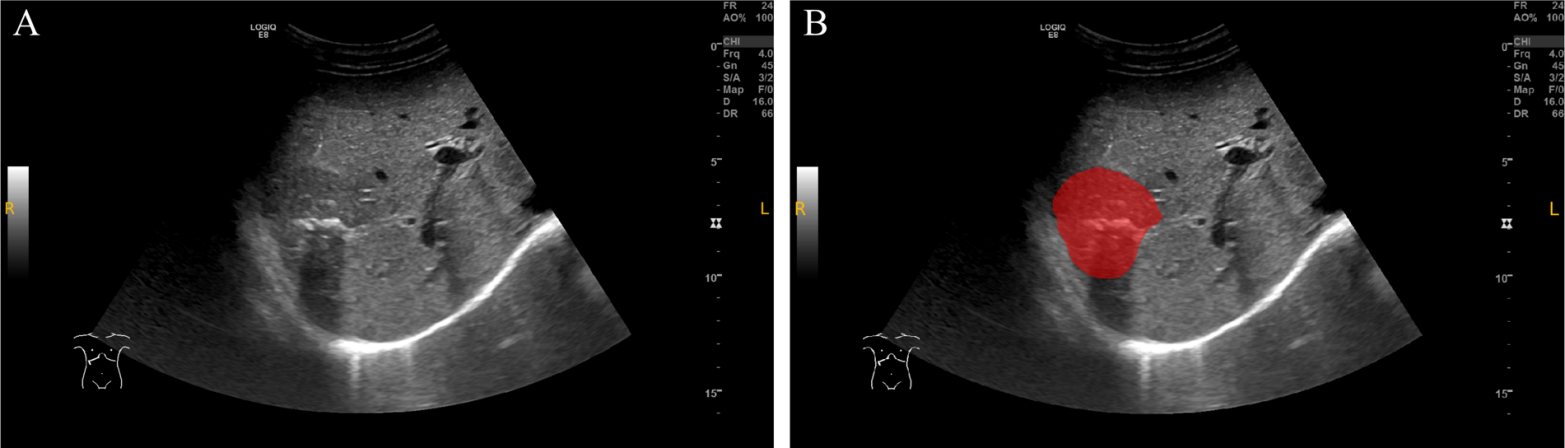
ROI delineation on ultrasound images. **(A)** Original grayscale ultrasound image of a patient with intrahepatic cholangiocarcinoma (ICC) combined with bile duct stones. **(B)** The region of interest (ROI) was manually delineated (red area) along the tumor margin. This case demonstrates the challenge in differentiating ICC with bile duct stones from intrahepatic bile duct stones with cholangitis (IBDS-IL) based on imaging alone.

The delineation of the region of interest (ROI) was performed by two ultrasound physicians using ITK-SNAP software (Version 4.0.0, http://www.itksnap.org) (16). The two radiologists independently outlined the ROIs along the tumor boundaries without access to clinical data, and then repeated the ROI delineation on the same patient’s ultrasound images one week later to assess inter-observer and intra-observer consistency. The procedure steps were as follows: 1) The maximum slice of the lesion in DICOM format was imported into ITK-SNAP software and saved as a “NiFTI” format for further use; 2) The Polygon Mode was selected, and the ROI was carefully delineated along the tumor’s edge. Afterward, the Paintbrush Mode was used to make adjustments to ensure precise coverage of the lesion; 3) The ROI image was exported and saved in “NiFTI” format for subsequent analysis (**Figure 2**).

### 2.4 Feature extraction and dimension reduction

Before feature extraction, the images underwent a meticulous standardization process to ensure uniformity and consistency across the dataset: resampling the images to achieve a consistent spatial resolution of 3 × 3 × 3 mm³, normalizing intensity values to 32 gray levels using a scale of 255, and effectively removing machine-specific artifacts or noise. After aligning the tumor’s maximum slice with the ROI’s NiFTI images, feature extraction was performed using the open-source radiomics toolkit PyRadiomics. Extracted features included shape features, first-order statistical features, and texture features. Additionally, image filtering techniques (such as wavelet, square, square root, logarithm, exponential, gradient, and local binary patterns) were applied to the original images. Features including first-order statistics and texture features were also extracted from the filtered images. After feature extraction, the data were standardized using Z-score normalization.

After feature extraction, the reliability of the features was assessed using intra-class correlation coefficient analysis. Both intra-observer and inter-observer intra-class correlation coefficients were calculated to evaluate the consistency of the extracted features. Features with an intra-class correlation coefficient exceeding 0.8 were deemed reliable and selected for further analysis.

To further streamline the dataset, a comprehensive dimensionality reduction process was carried out. Initially, features with high collinearity (correlation coefficient > 0.75) were removed to eliminate redundancy and multicollinearity. This was followed by a t-test to identify features with significant differences between groups (*p*-value < 0.05), ensuring the retention of statistically relevant features. Next, least absolute shrinkage and selection operator (LASSO) regression was applied to shrink and select key features by penalizing less important variables. Finally, recursive feature elimination (RFE) was employed to rank and iteratively eliminate less important features. This multi-step approach effectively reduced the dimensionality of the dataset while retaining the most predictive features for further model development.

### 2.5 Model construction and evaluation

The predictive models were developed in three components: the radiomics model, the clinical model, and the combined model. For the radiomics model, multiple machine learning algorithms were utilized, and the optimal hyperparameters were identified through a combination of Random Search and Grid Search to ensure optimal performance. The clinical model was constructed by including variables that demonstrated significant differences (*p*-value < 0.05) between ICC and IBDS-IL in the training set. These variables were first screened using univariate logistic regression, followed by multivariate logistic regression to build the final clinical model. Lastly, the combined model was created by integrating the best-performing radiomics model with the clinical model, aiming to harness the strengths of both approaches for enhanced predictive capability.

### 2.6 Statistical analysis

All radiomics procedures and statistical analyses were conducted using Python (Version 3.10), while R software (Version 4.3.1, R Foundation for Statistical Computing, Vienna, Austria) was used for constructing the nomogram and generating calibration curves. Continuous variables were reported as mean ± standard deviation or median (range), depending on the data distribution. Group comparisons for continuous variables were performed using the t-test or Mann-Whitney U test, as appropriate. Model performance differences were assessed using the DeLong test. Calibration curves were employed to evaluate the agreement between predicted and observed outcomes. Additionally, decision curve analysis (DCA) was carried out to assess the clinical utility of the models. A two-tailed *p*-value < 0.05 was considered statistically significant for all analyses.

## 3 Results

### 3.1 Patient characteristics

A total of 169 patients were included in the study, comprising 97 with IBDS-IL and 72 with ICC. The clinical characteristics of the patients, including comparisons between IBDS-IL and ICC, as well as between the training and validation groups, are summarized in **Table 1**. No significant differences in demographic or baseline clinical characteristics were observed between the training and validation groups (**Supplementary Material**), ensuring consistency for model development.

In the training set, several variables demonstrated significant differences between IBDS-IL and ICC. These included age, carcinoembryonic antigen (CEA), cancer antigen 125 (CA125), alanine aminotransferase (ALT), gender, presence of abdominal pain, combined bile duct stones, smoking status, alcohol consumption, history of other tumors, and liver atrophy (*p* < 0.05 for all).

### 3.2 Clinical model

Variables that exhibited significant differences between the IBDS-IL and ICC groups were analyzed using univariate logistic regression. Those with a *p*-value < 0.05 in the univariate analysis were subsequently included in a multivariate logistic regression to identify independent predictors. The final clinical model was constructed using abdominal pain and liver atrophy as the most significant predictors. The detailed results of the univariate and multivariate logistic regression analyses are presented in **Table 2**.

**Table 2:** Univariate and multivariate logistic analysis of clinical factors.

Variables	Univariate logistic analysis results		Multivariate logistic analysis results	
	*OR* (95% *CI*)	*p*-value	*OR* (95% *CI*)	*p*-value
Age	1.068 (1.022 - 1.116)	0.004^*^	1.056 (0.987 - 1.129)	0.111
CEA	1.567 (1.247 - 1.970)	<0.05^*^	1.376 (0.927 - 2.043)	0.113
CA125	1.033 (1.012 - 1.054)	0.002^*^	1.027 (0.994 - 1.061)	0.107
ALT	0.995 (0.991 - 1.000)	0.029^*^	0.999 (0.992 - 1.006)	0.770
Gender	3.230 (1.510 - 6.905)	0.002^*^	0.815 (0.155 - 4.294)	0.810
Abdominal Pain	0.079 (0.033 - 0.191)	<0.05^*^	0.178 (0.040 - 0.781)	0.022^*^
Combined with Bile Duct Stones	0.188 (0.081 - 0.434)	<0.05^*^	0.362 (0.075 - 1.742)	0.205
Smoking	3.973 (1.406 - 11.229)	0.009^*^	0.650 (0.053 - 8.010)	0.737
Drinking Alcohol	3.973 (1.406 - 11.229)	0.009^*^	6.910 (0.782 - 61.043)	0.082
Combined with Other Tumors	3.177 (1.226 - 8.230)	0.017^*^	1.443 (0.275 - 7.562)	0.665
Liver Atrophy	0.065 (0.026 - 0.161)	<0.05^*^	0.067 (0.016 - 0.279)	<0.05^*^

CEA, carcinoembryonic antigen; CA125, cancer antigen 125; ALT, alanine transaminase; *p<0.05.

**Figure 3:**
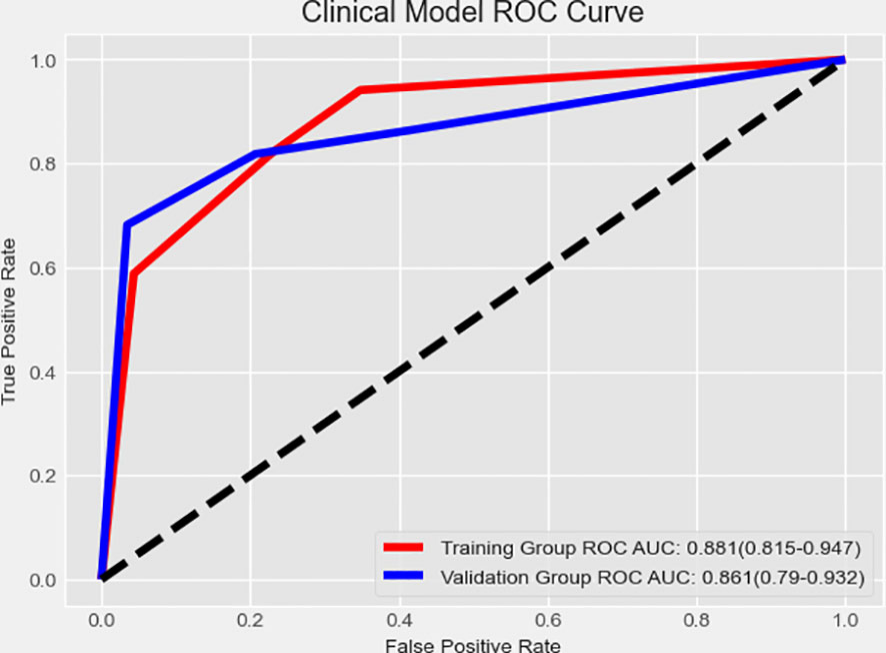
Receiver operating characteristic curve analysis of the clinical model.

The clinical model demonstrated strong predictive performance, achieving an AUC of 0.881 (0.815–0.947) in the training group and 0.861 (0.79–0.932) in the validation group (**Figure 3**).

### 3.3 Radiomics model

Prior to constructing the radiomics model, an extensive feature reduction process was implemented to minimize the risk of overfitting. Initially, 1431 features were extracted from both the original and filtered images. Intra-observer reliability, as measured by the intra-class correlation coefficient, exceeded 0.8 for all features, while 1376 features demonstrated an intra-class correlation coefficient greater than 0.8 for inter-observer reliability, indicating strong consistency.

To refine the feature set, several key steps were undertaken. First, features exhibiting high collinearity (correlation > 0.75) were removed to reduce multicollinearity. Next, t-tests were performed to identify significant features, followed by LASSO regression with 10-fold cross-validation for further dimensionality reduction. Detailed information on the LASSO process and cross-validation results can be found in the **Supplementary Materials**.

**Figure 4:**
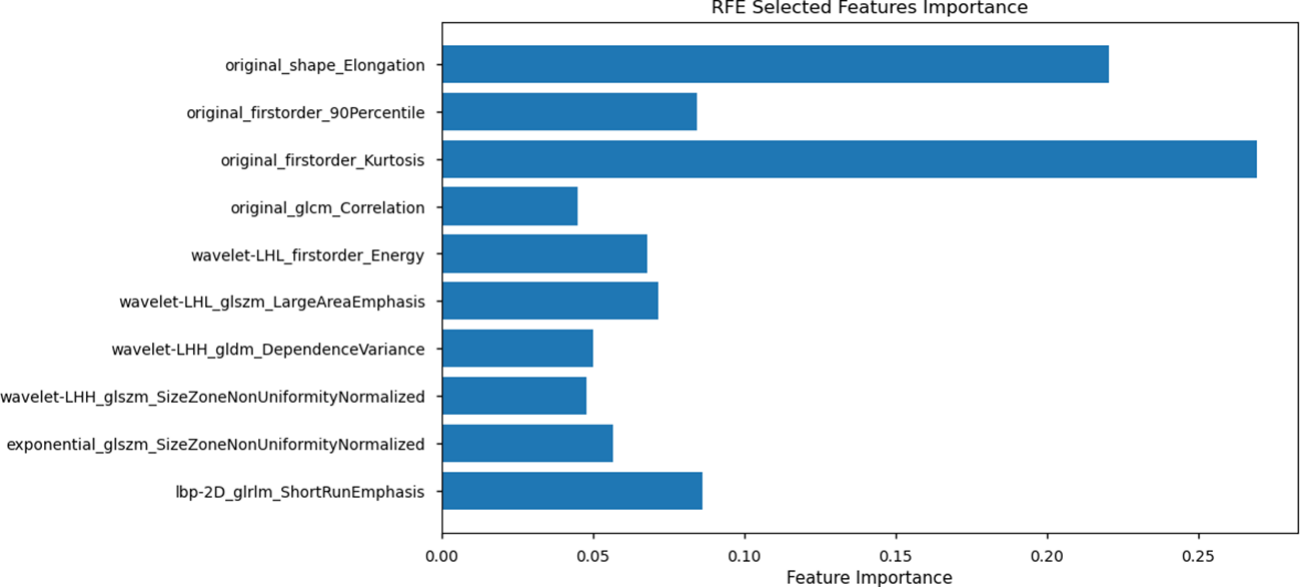
Recursive Feature Elimination (RFE) selected feature importance. The plot displays the top 10 features selected using RFE with Random Forest as the evaluation model. Feature importance values are represented along the x-axis, with individual features listed on the y-axis.

Despite retaining 16 features after these steps, the model still exhibited a potential risk of overfitting, owing to the relatively small sample size (n = 118) in the training set. To address this issue, RFE was applied, using Random Forest as the evaluation model to select the top ten most significant features for the final model (**Figure 4**).

**Figure 5:**
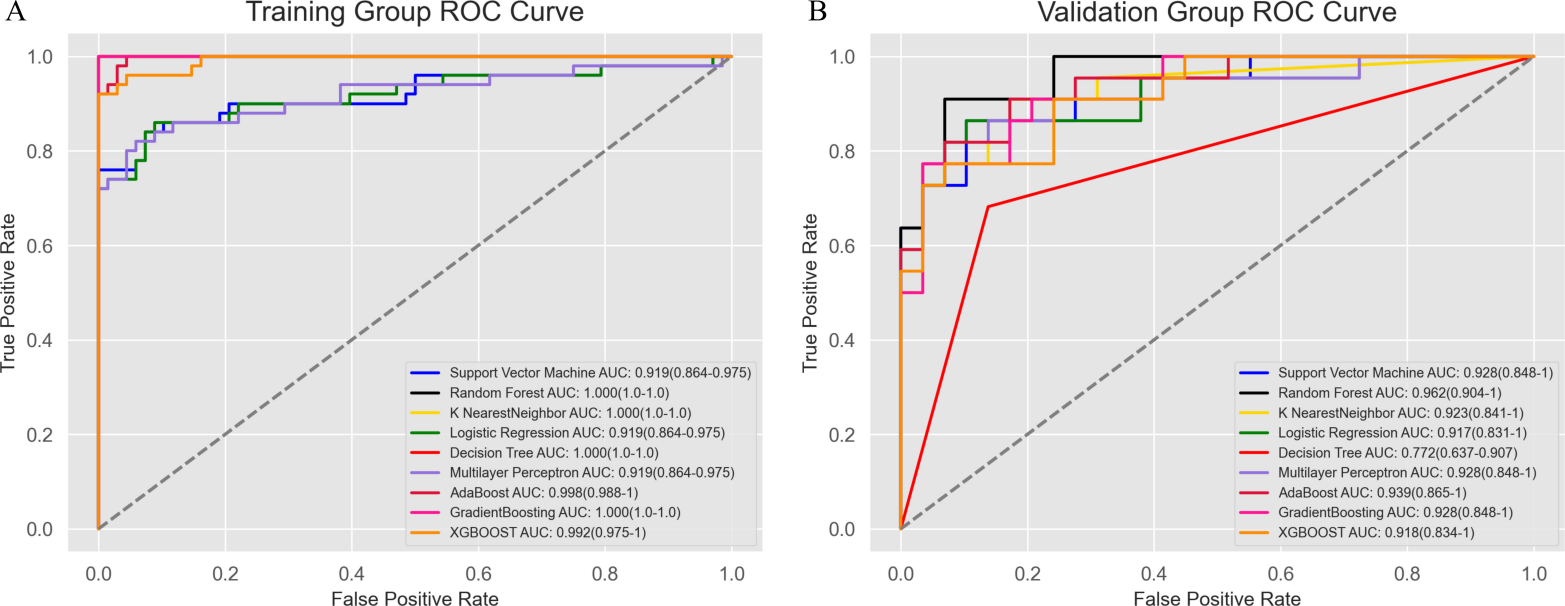
Receiver operating characteristic curve analysis of the modeling methods. The Random Forest model showed the best diagnostic performance, with AUC values of 1.0 (1.0–1.0) in the training group **(A)** and 0.962 (0.904–1) in the validation group **(B)**.

To construct the radiomics model, we explored a variety of machine learning algorithms, including Support Vector Machine, Random Forest, K-Nearest Neighbor, Logistic Regression, Decision Tree, Artificial Neural Network, AdaBoostClassifier, GradientBoostingClassifier, and XGBoost. Both RandomizedSearchCV and GridSearchCV were employed to identify the optimal hyperparameters for each algorithm, ensuring the best possible model performance (the specific optimal parameters are detailed in the **Supplementary Materials**). ROC curves were plotted to evaluate the performance of the models, and the AUC was calculated (**Figure 5**). Among all the algorithms tested, the Random Forest model achieved the highest AUC of 0.962 (0.904-1), demonstrating its superior predictive ability.

Although the AUC of the best-performing radiomics model was higher than that of the clinical model, the DeLong test revealed no statistically significant difference between the AUCs of the radiomics model (Random Forest, 0.962) and the clinical model (0.861; *p* = 0.111). This suggests that both models demonstrate comparable predictive accuracy.

### 3.4 Combined model

**Figure 6:**
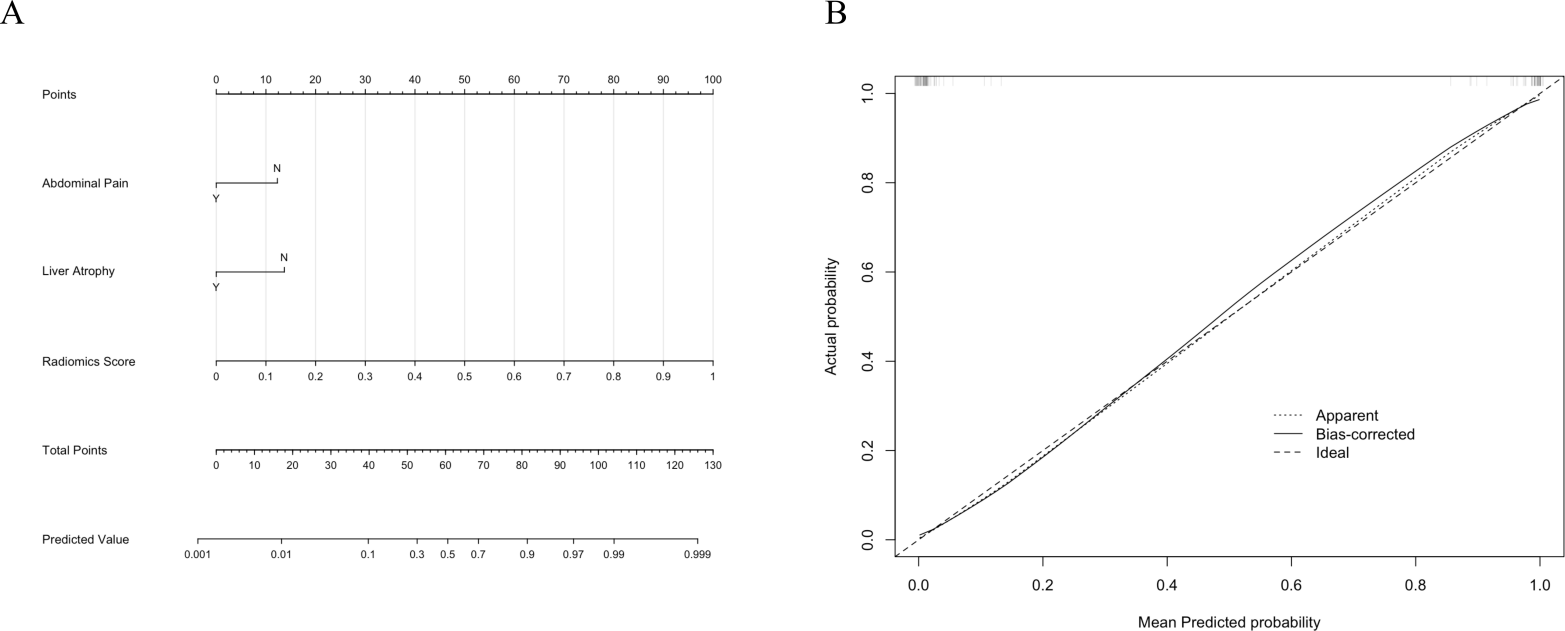
**(A)** The nomogram for the combined model integrates clinical factors (Abdominal Pain and Liver Atrophy) and the Radiomics Score to predict the probability of intrahepatic bile duct stones with cholangitis (IBDS-IL) and intrahepatic cholangiocarcinoma (ICC). **(B)** Calibration curve of the combined model. The dotted line represents the apparent performance, the solid line indicates the bias-corrected results, and the dashed line represents the ideal performance.

The combined model was constructed by integrating the predicted values of the best-performing radiomics model (Random Forest) as the Radiomics Score with the clinical model. This integrated model was visualized using a nomogram (**Figure 6A**), which provides an intuitive tool for predicting individual probabilities based on the combined model.

To assess the calibration of the combined model, a calibration curve was plotted (**Figure 6B**). The curve demonstrated excellent agreement between predicted and observed outcomes, indicating the reliability of the model’s predictions. Additionally, the Hosmer-Lemeshow test yielded a *p*-value of 0.998, confirming that there was no significant deviation from a perfect fit.

**Figure 7:**
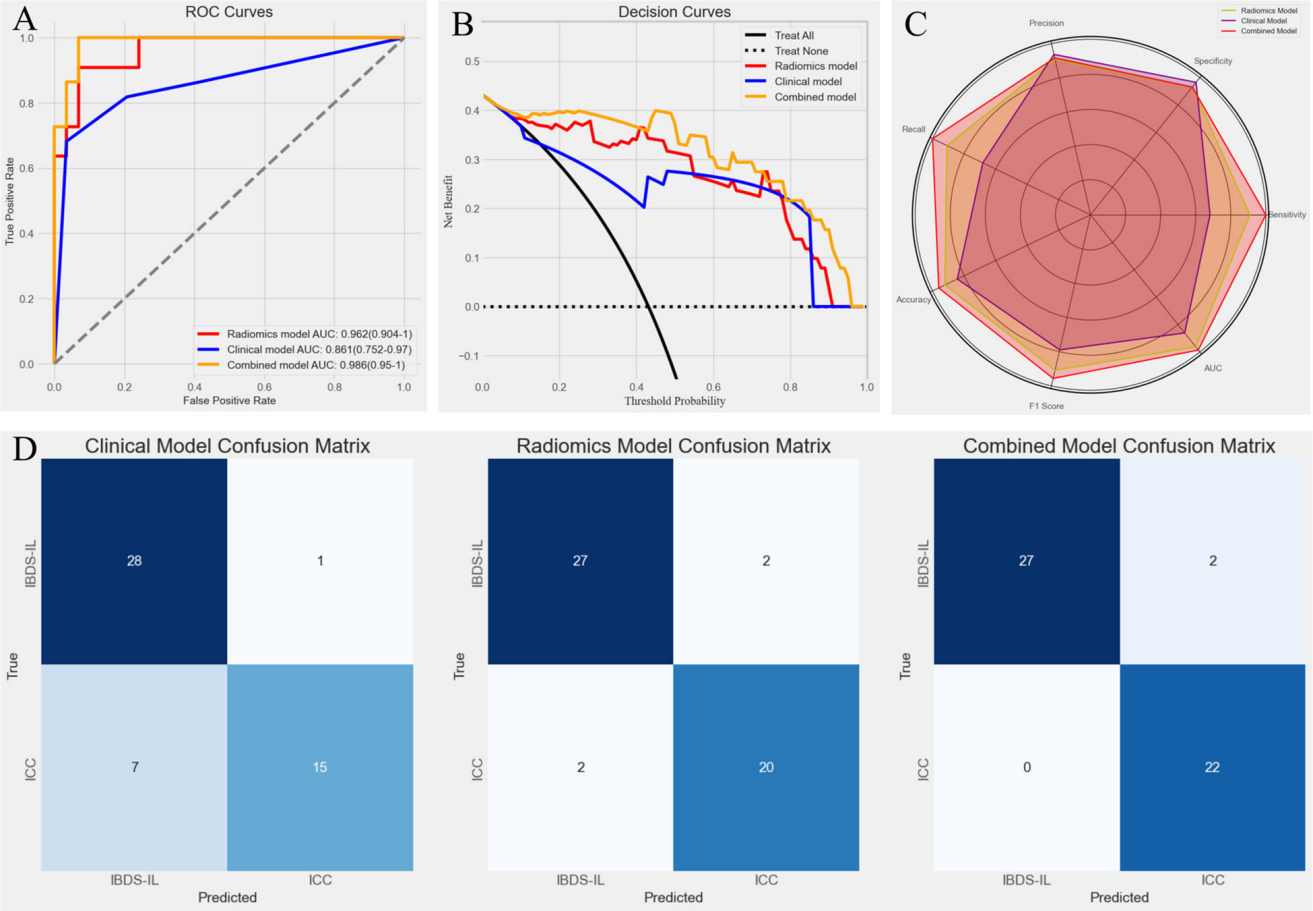
**(A)** Receiver Operating Characteristic (ROC) curves for the three models. The combined model achieved the highest AUC (0.988), followed by the radiomics model (0.962) and the clinical model (0.861), demonstrating superior predictive performance of the combined model. **(B)** Decision Curve Analysis (DCA) for the three models. The combined model (yellow line) provided the greatest net benefit across a wide range of threshold probabilities, indicating its superior clinical utility compared to the radiomics model (red line) and clinical model (blue line). **(C)** Radar chart comparing key performance metrics (precision, specificity, sensitivity, AUC, F1 score, accuracy, and recall) for the three models. **(D)** Confusion matrices for the clinical model, radiomics model, and combined model. The combined model showed the best classification performance, with fewer misclassifications, particularly in identifying ICC cases (0 misclassified).

The predictive performance of the combined model was evaluated alongside the radiomics and clinical models using ROC curves (**Figure 7A**). The combined model achieved the highest AUC of 0.988 (0.967–1), significantly outperforming the clinical model (*p* < 0.05, DeLong test) but showing no statistically significant difference compared to the radiomics model. To further illustrate the models’ clinical utility, DCA was performed (**Figure 7B**). The combined model demonstrated the greatest net benefit across a wide range of threshold probabilities, indicating its superior value in guiding clinical decision-making. To provide a comprehensive evaluation of the models, a radar chart (**Figure 7C**) was generated to compare key metrics, including precision, specificity, sensitivity, AUC, F1 score, accuracy, and recall. The combined model consistently outperformed the other models across these metrics, further highlighting its predictive and clinical effectiveness.

Finally, confusion matrices were generated for all three models (**Figure 7D**), providing a detailed visualization of their classification performance. The results clearly demonstrated the superiority of the combined model, which achieved the highest accuracy with no misclassified ICC cases. In contrast, the clinical model showed a tendency to misclassify ICC as IBDS-IL, which may have significant clinical implications. The radiomics model performed better but still resulted in minor misclassifications. The combined model’s ability to completely avoid misclassifying ICC highlights its potential clinical value in ensuring accurate diagnosis and timely intervention.

**Figure 8:**
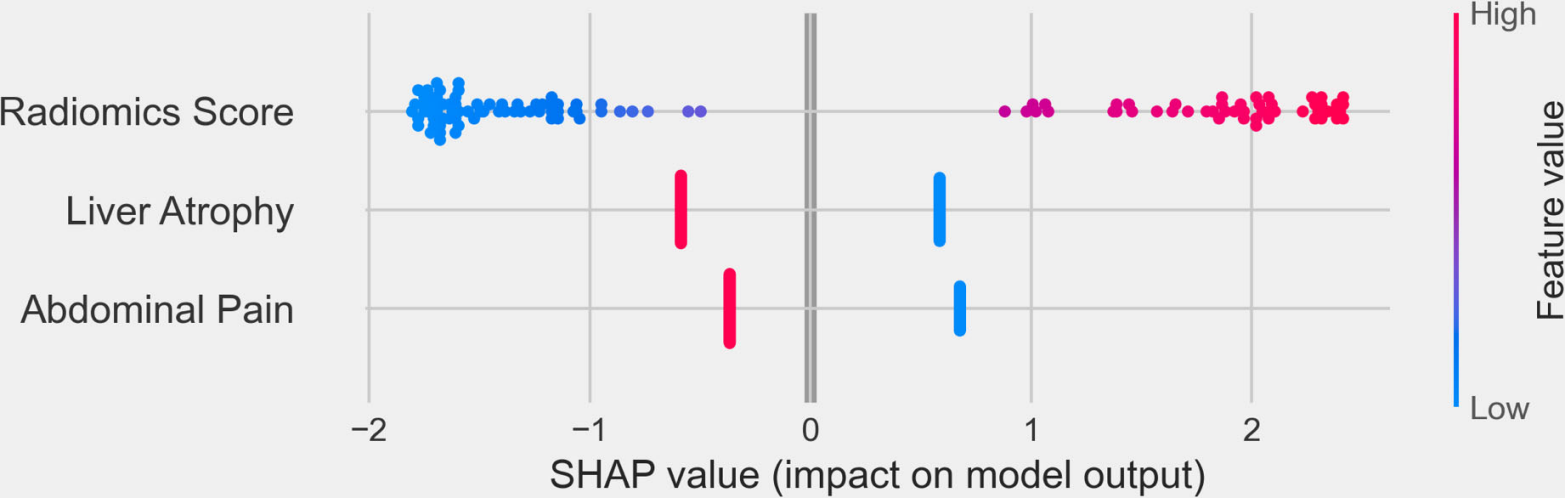
SHapley Additive exPlanations (SHAP) summary plot illustrating the contribution of individual features to the combined model’s output. The x-axis represents the SHAP values, reflecting the impact of each feature on the model’s predictions. Positive SHAP values indicate a higher likelihood of predicting ICC, while negative SHAP values correspond to IBDS-IL.

To further interpret the combined model, we performed SHapley Additive exPlanations (SHAP) analysis to quantify the contribution of each feature to the model’s predictions. The SHAP summary plot (**Figure 8**) revealed that the Radiomics Score was the most significant contributor to the model, indicating that the radiomics features played a dominant role in distinguishing IBDS-IL from ICC. Other clinical factors, including abdominal pain and liver atrophy, also contributed to the model but to a lesser extent.

### 3.5 Bootstrap validation

To further validate the robustness and reliability of all predictive models, including the radiomics model, clinical model, and combined model, we performed a bootstrap analysis with 1000 resamples for each model. The bootstrap-derived AUC and 95% confidence intervals were highly consistent with those obtained using the original validation group (**Supplementary Materials**), confirming the stability and reliability of each model’s predictive performance. These findings further demonstrate the robustness of the combined model, as well as the radiomics and clinical models, across different evaluation methods.

## 4 Discussion

To the best of our knowledge, our study is the first to integrate ultrasound radiomics features with clinical characteristics for the preoperative differentiation of IBDS-IL and ICC. By combining the strengths of radiomics and clinical data, the proposed combined model achieved outstanding predictive performance, with an AUC of 0.988, significantly outperforming the clinical model and demonstrating comparable accuracy to the radiomics model. This novel approach highlights the added value of integrating imaging-based features, which capture subtle tumor characteristics, with clinical variables that reflect patient-specific factors. The visualization of the combined model using a nomogram provides an intuitive tool for individualized risk prediction, facilitating its application in clinical practice. Additionally, the calibration curve and DCA demonstrated not only the reliability of the model’s predictions but also its substantial clinical utility, underscoring the potential of this approach to improve preoperative decision-making and patient management.

In this study, we chose ultrasound radiomics over contrast-enhanced imaging modalities due to its unique advantages in clinical practice. Ultrasound is widely accessible, cost-effective, and non-invasive, making it a practical tool for routine clinical use, especially in resource-limited settings (17, 18). Furthermore, it provides real-time, dynamic imaging of biliary structures, offering unique insights into lesion characteristics that are not readily captured by other imaging modalities (19, 20). While contrast-enhanced CT or MRI can offer valuable diagnostic information, these modalities are not always available and their diagnostic accuracy for distinguishing IBDS-IL complicated by ICC remains limited, as shown in previous studies (21). Our primary objective was to enhance the diagnostic utility of ultrasound, which is already a first-line imaging modality for biliary diseases. By applying radiomics analysis to ultrasound, we sought to overcome the limitations of conventional ultrasound techniques and improve diagnostic accuracy in a widely accessible manner.

During the construction of the clinical model, abdominal pain and liver atrophy were ultimately included as key predictors, while traditional tumor biomarkers such as CA199 and CEA were excluded. CA199 is known to be easily influenced by inflammation, which likely compromises its specificity in differentiating IBDS-IL from ICC (22, 23). However, the exclusion of CEA, a biomarker typically regarded as more specific for malignancy, warrants further discussion (24). CEA has long been associated with various gastrointestinal malignancies, including cholangiocarcinoma, and is considered a useful marker for cancer diagnosis and prognosis (25). Its lack of significance in this study may be due to several factors. First, the overlap in CEA levels between early-stage ICC and benign conditions such as IBDS-IL could reduce its discriminatory power (26). Second, our study population consisted exclusively of surgical candidates, where CEA levels may not differ significantly between groups due to the early or resectable stage of the disease (27). Third, the relatively small sample size may have limited the statistical power to detect CEA’s potential contribution. The findings in our study suggest that the predictive value of CEA in this specific context may be limited, particularly in distinguishing between IBDS-IL and early-stage ICC in surgical candidates, highlighting the importance of considering the clinical and pathological context when interpreting biomarker significance.

The clinical model, with an AUC of 0.861, demonstrated moderate predictive performance, which reflects its reliance on observable clinical features such as abdominal pain and liver atrophy. While these features provide valuable diagnostic insights, they may lack sensitivity in distinguishing subtle differences between IBDS-IL and ICC, particularly in early or resectable stages. In comparison, the radiomics model achieved a higher AUC of 0.962, highlighting its ability to capture imaging-derived microstructural and textural features that are difficult to assess clinically (28, 29). These features provide a deeper understanding of the tumor’s biological and morphological characteristics, offering a distinct advantage in differentiating between IBDS-IL and ICC. However, despite its high accuracy, the radiomics model lacks the contextual information provided by clinical data, which can be crucial for practical decision-making (30, 31). The combined model demonstrated the highest AUC of 0.988, significantly outperforming the clinical model and showing comparable performance to the radiomics model. This improvement can be attributed to the integration of complementary data sources, where radiomics features provide high sensitivity for subtle imaging patterns, and clinical data enhance the model’s interpretability and applicability in clinical practice (32).

Furthormore, the results of the confusion matrices highlight the clinical significance of the combined model, particularly in addressing the limitations of the clinical model. The clinical model showed a tendency to misclassify ICC cases as IBDS-IL, which could have serious implications for patient prognosis. Missing an ICC diagnosis may delay appropriate surgical treatment and lead to disease progression, significantly affecting patient outcomes (33). In contrast, the combined model demonstrated 100% accuracy in identifying ICC cases, with no misclassifications. This achievement underscores the importance of integrating radiomics features with clinical variables. The radiomics score, as indicated by the SHAP analysis, played a dominant role in the combined model by providing imaging-based insights that effectively distinguish between the two conditions. By reducing the risk of misdiagnosis, the combined model not only improves diagnostic accuracy but also holds significant clinical value in ensuring timely and appropriate intervention for ICC patients. We believe this improvement could have a profound impact on patient management, particularly in guiding surgical decision-making and optimizing treatment strategies.

The integration of radiomics features with clinical variables further strengthened the model’s predictive capability while demonstrating superior clinical utility, as supported by the DCA and SHAP analysis results. The DCA showed that the combined model provided the greatest net benefit across a wide range of threshold probabilities, reinforcing its potential value in guiding clinical decision-making. This indicates that the combined model can offer more accurate risk stratification and better inform treatment decisions compared to the radiomics or clinical models alone. SHAP analysis further illuminated the contribution of individual features to the combined model. Among all features, the Radiomics Score emerged as the most significant contributor, underscoring the dominant role of radiomics in capturing imaging-based characteristics critical for differentiating IBDS-IL from ICC. Clinical features, such as abdominal pain and liver atrophy, also contributed to the model’s predictions, albeit to a lesser extent.

Although the combined model demonstrated excellent predictive performance, its integration into clinical practice remains a significant challenge and is far from being realized. The current model is research-oriented and has not yet been validated in real-world clinical workflows or for complex cases, such as patients presenting with both IBDS-IL and malignant transformation (e.g., coexisting IBDS-IL and ICC). In this study, such cases were classified into the ICC group, as malignant transformation is the primary clinical concern due to its prognostic and therapeutic implications. However, the limited sample size prevented separate validation for this specific scenario, which remains a potential limitation. Future research should focus on validating the model with larger, multicenter datasets and evaluating its performance in more nuanced contexts. Additionally, prospective studies are needed to explore its clinical feasibility, including embedding predictive outputs like the Radiomics Score into clinical systems such as picture archiving and communication systems or electronic health records. Substantial efforts will be required to optimize the model’s efficiency, interpretability, and integration into clinical workflows to bridge the gap between research and practical application, ultimately improving patient management and outcomes.

Despite the superior performance of the combined model, several limitations of this study should be addressed. First, the sample size was relatively small, particularly for a study employing machine learning methods. A limited sample size can introduce potential bias and reduce the statistical power of the results, potentially affecting the stability of feature selection and the generalizability of the model. Additionally, small datasets increase the risk of overfitting, where the model may perform well on the training data but struggle to generalize to unseen data. To address these concerns, future studies should consider expanding the dataset by incorporating multi-center data or collecting additional cases from diverse populations. Such efforts would not only enhance the statistical power but also improve the robustness and reproducibility of the model. Independent validation using external datasets is also essential for evaluating the model’s applicability in different clinical settings. Second, this study focused exclusively on surgical candidates, introducing a potential selection bias that may limit the applicability of the findings to patients with more advanced disease stages who are not eligible for surgery. Future research should aim to address these limitations by including a more diverse patient population and incorporating external validation with datasets from multiple centers. Third, Abdominal pain and liver atrophy were included as clinical predictors of ICC in this study. While statistically significant, these variables are not strictly objective. Abdominal pain was recorded based on patient reports and physician documentation without standardized severity grading, introducing potential variability. Liver atrophy was qualitatively assessed by radiologists based on imaging findings rather than precise volumetric measurements. Future studies should incorporate standardized pain scoring systems and quantitative imaging analysis to improve reproducibility and minimize subjectivity in clinical assessments. Additionally, integrating other data modalities, such as genomic or molecular profiling, may further enhance the predictive power and clinical utility of the model. These steps will help refine the combined model and facilitate its translation into routine clinical practice.

## 5 Conclusion

In summary, our study demonstrates that integrating radiomics features with clinical variables significantly enhances the preoperative differentiation of IBDS-IL and ICC. The inclusion of clinically relevant features, such as abdominal pain and liver atrophy, alongside imaging-derived radiomics scores, underscores the importance of a multimodal approach in disease differentiation. Moreover, the combined model demonstrated excellent calibration and substantial clinical utility, making it a promising tool for clinical decision-making. However, further validation with larger, multicenter datasets and inclusion of diverse patient populations is necessary to confirm its robustness and generalizability.

## Data Availability

The original contributions presented in the study are included in the article/**Supplementary Material**. Further inquiries can be directed to the corresponding author.
