# Supplementary material for: A combined radiomics and clinical model for preoperative differentiation of intrahepatic cholangiocarcinoma and intrahepatic bile duct stones with cholangitis: a machine learning approach
Source: Front Oncol. 2025 Mar 17;15:1546940. doi: 10.3389/fonc.2025.1546940 (PMC11955465; doi:10.3389/fonc.2025.1546940)
Supplement: Supplementary file 1 [file DataSheet1.docx]

1. **Ultrasound machines**

Ultrasound examination was performed by using one of the following ultrasound machines: LOGIQ E8 (GE Healthcare, United States; C5-1 convex array probes, 1–5 MHz); LOGIQ E9 (GE Healthcare, United States; C5-1 convex array probes, 1–5 MHz); Aplio 500 (Toshiba Medical systems, Japan; 6C1 probe, 1–6 MHz); i800 (Cannon Medical systems Corporation, Japan; i8CX1 probe, 1-8MHz); and Resona 7T (Mindray, China; SC6-1 U probe, 1-6MHz).

1. **Comparison of clinical characteristics between the training and validation groups**

| **Variables** | **Training Group(n=118)** | **Testing Group(n=51)** | **p** |
| --- | --- | --- | --- |
| **Age** | 64.65±9.51 | 64.53±9.53 | 0.939 |
| **BMI** | 22.2±2.98 | 22.2±2.81 | 0.997 |
| **AFP** | 4.6±19.41 | 20.2±123.56 | 0.186 |
| **CA199** | 2399.19±11469.56 | 1075.24±2125.05 | 0.417 |
| **CEA** | 13.35±43.3 | 12.35±55.13 | 0.9 |
| **CA125** | 72.32±225.03 | 93.18±459.63 | 0.695 |
| **ALT** | 84.16±183.67 | 95.57±131.37 | 0.69 |
| **AST** | 104.9±329.51 | 85.56±129.51 | 0.687 |
| **ALP** | 162.58±154.02 | 161.42±124.14 | 0.963 |
| **GGT** | 197.38±297.42 | 191.45±276.94 | 0.904 |
| **TBIL** | 31.41±74.35 | 22.95±23.25 | 0.429 |
| **DBIL** | 17.41±56.72 | 9.65±15.81 | 0.341 |
| **ALB** | 37.53±5.22 | 37.84±5.04 | 0.724 |
| **PT** | 12.63±1.32 | 12.63±1.2 | 0.982 |
| **INR** | 1.04±0.11 | 1.03±0.1 | 0.956 |
| **Gender** |  |  | 0.553 |
| **Female** | 66 | 26 |  |
| **Male** | 52 | 25 |  |
| **Abdominal Pain** |  |  | 0.328 |
| **N** | 46 | 24 |  |
| **Y** | 72 | 27 |  |
| **Combined with Common Bile Duct Stones** |  |  | 0.486 |
| **N** | 72 | 34 |  |
| **Y** | 46 | 17 |  |
| **Weight Loss in the Past 3 Months** |  |  | 0.539 |
| **N** | 117 | 50 |  |
| **Y** | 1 | 1 |  |
| **Smoking** |  |  | 0.382 |
| **N** | 95 | 38 |  |
| **Y** | 23 | 13 |  |
| **Drinking Alcohol** |  |  | 0.063 |
| **N** | 100 | 37 |  |
| **Y** | 18 | 14 |  |
| **Diabetes** |  |  | 0.617 |
| **N** | 105 | 44 |  |
| **Y** | 13 | 7 |  |
| **Hypertension** |  |  | 0.54 |
| **N** | 82 | 33 |  |
| **Y** | 36 | 18 |  |
| **Hepatitis B** |  |  | 0.298 |
| **N** | 108 | 44 |  |
| **Y** | 10 | 7 |  |
| **Combined with Other Tumors** |  |  | 0.186 |
| **N** | 97 | 46 |  |
| **Y** | 21 | 5 |  |
| **Family History of Tumors** |  |  | 0.35 |
| **N** | 116 | 51 |  |
| **Y** | 2 | 0 |  |
| **Liver Atrophy** |  |  | 0.292 |
| **N** | 59 | 21 |  |
| **Y** | 59 | 30 |  |
| **Liver Cirrhosis** |  |  | 0.942 |
| **N** | 106 | 46 |  |
| **Y** | 12 | 5 |  |
| **Fatty Liver** |  |  | 0.758 |
| **N** | 106 | 45 |  |
| **Y** | 12 | 6 |  |

BMI, body mass index; AFP, alpha fetoprotein; CA199, cancer antigen 199; CEA, carcinoembryonic antigen; CA125, cancer antigen 125; ALT, alanine transaminase; AST, aspartate transaminase; ALP, alkaline phosphatase; GGT, Gamma-Glutamyl transferase; TBIL, total bilirubin; DBIL, directed bilirubin; ALB, albumin level; PT, prothrombin time; INR, international normalized ratio.

1. **LASSO procedure**

**
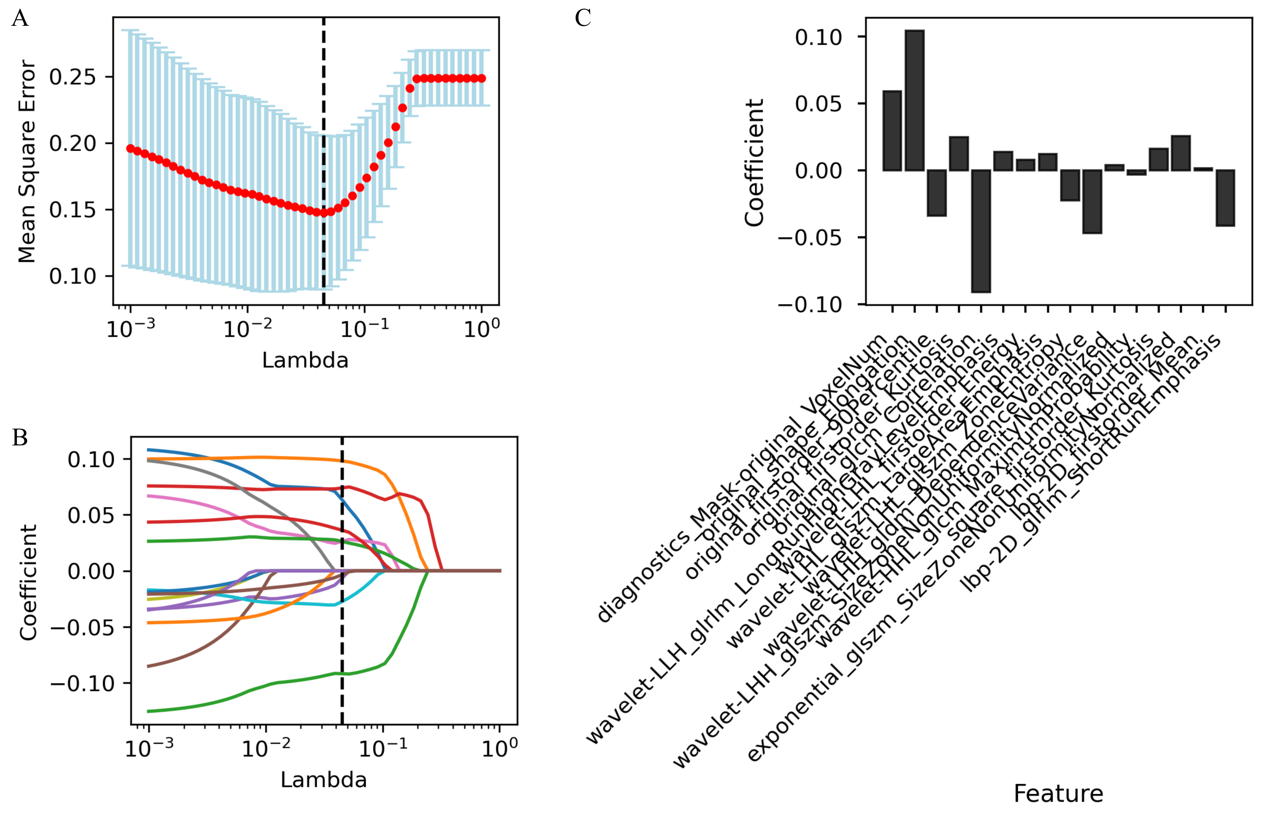
**

**Fig S1.** (A, B) Least absolute shrinkage and selection operator (LASSO) regression complexity was controlled using a tuning parameter lambda; the optimal lambda value for the minimized mean squared error was 0.044984326689694466. (C) The 16 selected variables and their coefficient values are shown.

1. **The LASSO selected radiomics features and their coefficient values**

| **Filter** | **Feature class** | **Feature** | **Coefficient** |
| --- | --- | --- | --- |
| diagnostics | Mask-original | VoxelNum | 0.059216693 |
| original | shape | Elongation | 0.104638685 |
| original | firstorder | 90Percentile | -0.033916021 |
| original | firstorder | Kurtosis | 0.024988221 |
| original | glcm | Correlation | -0.090948813 |
| wavelet-LLH | glrlm | LongRunHighGrayLevelEmphasis | 0.013681398 |
| wavelet-LHL | firstorder | Energy | 0.008030704 |
| wavelet-LHL | glszm | LargeAreaEmphasis | 0.012257796 |
| wavelet-LHL | glszm | ZoneEntropy | -0.022439348 |
| wavelet-LHH | gldm | DependenceVariance | -0.046774243 |
| wavelet-LHH | glszm | SizeZoneNonUniformityNormalized | 0.003781639 |
| wavelet-HHL | glcm | MaximumProbability | -0.003081253 |
| square | firstorder | Kurtosis | 0.01621899 |
| exponential | glszm | SizeZoneNonUniformityNormalized | 0.025693877 |
| lbp-2D | firstorder | Mean | 0.001457577 |
| lbp-2D | glrlm | ShortRunEmphasis | -0.04130792 |

1. **Best RandomSearch parameters in the radiomics model**

| **Model** | **Best RandomSearch Parameters** |
| --- | --- |
| **Support Vector Machine** | {'kernel': 'linear', 'gamma': 30.538555088334185, 'C': 0.04328761281083059} |
| **RandomForest** | {'n_estimators': 300, 'min_samples_split': 2, 'min_samples_leaf': 2, 'max_depth': None} |
| **K-Nearest Neighbor** | {'weights': 'distance', 'p': 1, 'n_neighbors': 3} |
| **LogisticRegression** | {'penalty': 'l2', 'C': 0.1} |
| **DecisionTree** | {'min_samples_split': 2, 'min_samples_leaf': 4, 'max_depth': 20} |
| **MLPClassifier** | {'learning_rate': 'constant', 'hidden_layer_sizes': (30, 20, 10), 'alpha': 1e-05, 'activation': 'logistic'} |
| **AdaBoostClassifier** | {'n_estimators': 100, 'learning_rate': 0.1} |
| **GradientBoostingClassifier** | {'n_estimators': 100, 'max_depth': 3, 'learning_rate': 1.0} |
| **XGBOOST** | {'n_estimators': 200, 'max_depth': 3, 'learning_rate': 0.01, 'gamma': 0.1} |

1. **Best GridSearch parameters in the radiomics model**

| **Model** | **Best GridSearch Parameters** |
| --- | --- |
| **Support Vector Machine** | {'C': 0.03895885152974753, 'gamma': 3.0538555088334185, 'kernel': 'linear'} |
| **RandomForest** | {'max_depth': None, 'min_samples_split': 2, 'n_estimators': 300} |
| **K-Nearest Neighbor** | {'n_neighbors': 3, 'p': 1, 'weights': 'distance'} |
| **LogisticRegression** | {'C': 0.02, 'penalty': 'l2'} |
| **DecisionTree** | {'max_depth': 20, 'min_samples_split': 2} |
| **MLPClassifier** | {'activation': 'logistic', 'alpha': 7.500000000000001e-05, 'hidden_layer_sizes': (30, 20, 10), 'learning_rate': 'constant'} |
| **AdaBoostClassifier** | {'learning_rate': 0.1, 'n_estimators': 150} |
| **GradientBoostingClassifier** | {'learning_rate': 1.0, 'max_depth': 3, 'n_estimators': 100} |
| **XGBOOST** | {'gamma': 0.1, 'learning_rate': 0.01, 'max_depth': 3, 'n_estimators': 250} |

1. **Bootstrap validation**


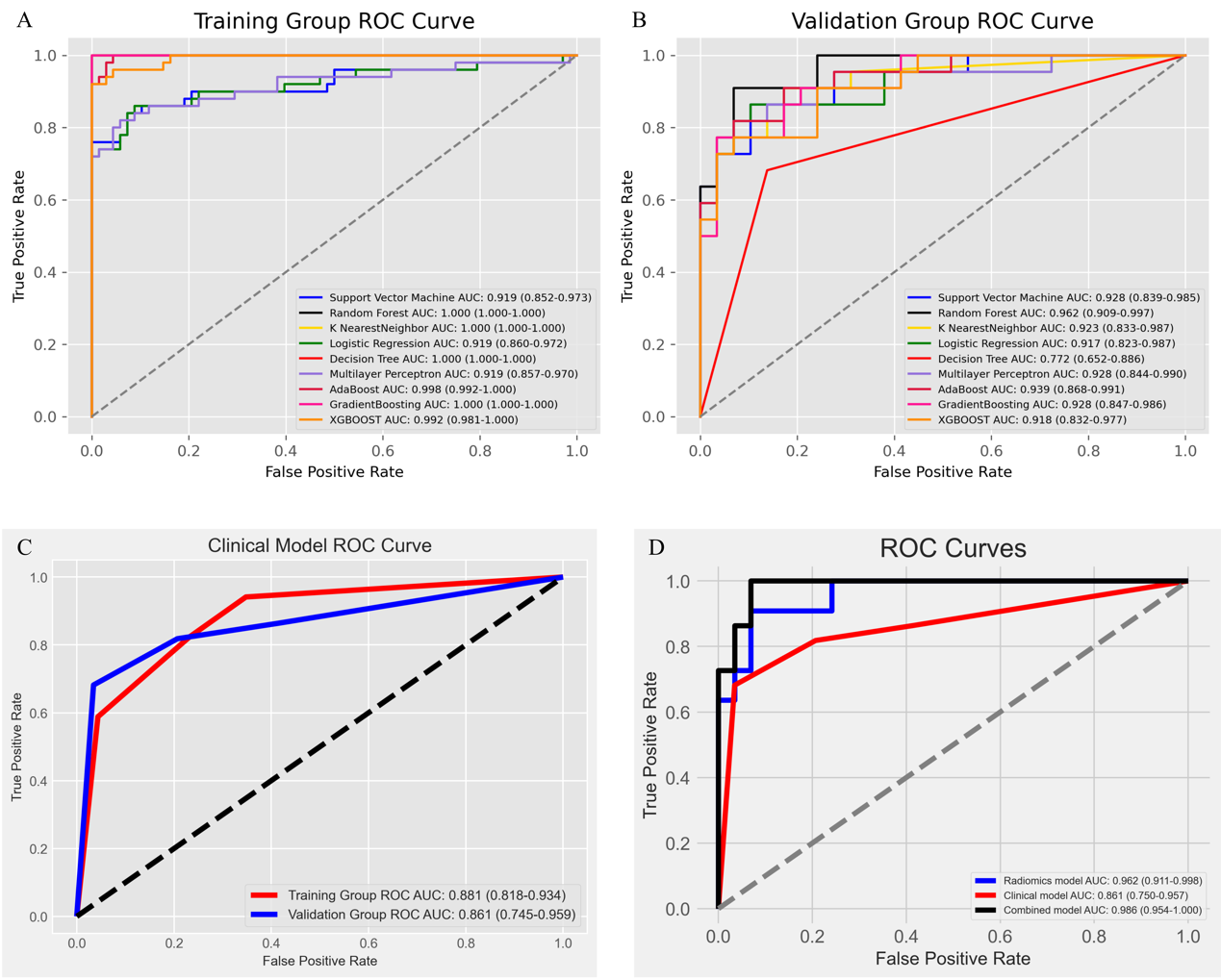


**Fig S2.** Receiver operating characteristic (ROC) curves for model validation after bootstrap analysis with 1000 resamples: (A) Radiomics model (training group); (B) Radiomics model (validation group); (C) Clinical model; (D) Combined model. The bootstrap-derived AUC and 95% confidence intervals for all models were highly consistent with the original results, confirming the robustness and reliability of the predictive performance.
